# Supplementary material for: Machine Learning Accelerates Crystallization for Structure Determination
Source: Angew Chem Int Ed Engl. 2026 May 4;65(25):e1218503. doi: 10.1002/anie.1218503 (PMC13266958; doi:10.1002/anie.1218503)

checkCIF/PLATON report

Structure factors have been supplied for datablock(s) lcz030

THIS REPORT IS FOR GUIDANCE ONLY. IF USED AS PART OF A REVIEW PROCEDURE FOR PUBLICATION, IT SHOULD NOT REPLACE THE EXPERTISE OF AN EXPERIENCED CRYSTALLOGRAPHIC REFEREE.

No syntax errors found. CIF dictionary Interpreting this report

Datablock: lcz030

Bond precision:	C-C = 0.0131 A		Wavelength=1.54184
Cell:	a=13.3303(1)	b=21.6652(3)	c=21.8805(3)
	alpha=106.904(1)	beta=105.988(1)	gamma=98.302(1)
Temperature:	100 K		

	Calculated	Reported
Volume	5636.51(13)	5636.51(12)
Space group	P -1	P -1
Hall group	-P 1	-P 1
Moiety formula	C35 H23 Ag3 F18 N6 O4, 2(C25 H13 Ag3 F18 N6 O2) [+ solvent]	1.5(C10 H2 Ag2 F12 N4), 1.333(C10 H10 O2)
Sum formula	C85 H49 Ag9 F54 N18 O8 [+ solvent]	C28.33 H16.33 Ag3 F18 N6 O2.67
Mr	3447.26	1149.08
Dx,g cm-3	2.031	2.031
Z	2	6
Mu (mm-1)	13.622	13.622
F000	3316.0	3316.0
F000’	3331.85	
h,k,lmax	16,27,27	16,27,27
Nref	24194	23072
Tmin,Tmax	0.113,0.149	0.626,1.000
Tmin’	0.050	

```
Correction method= # Reported T Limits: Tmin=0.626 Tmax=1.000
AbsCorr = MULTI-SCAN

Data completeness= 0.954          Theta(max)= 78.349

R(reflections)= 0.0714( 19185)          wR2(reflections)= 0.1871( 23072)

S = 1.096                 Npar= 1562
```

The following ALERTS were generated. Each ALERT has the format
test-name_ALERT_alert-type_alert-level.
Click on the hyperlinks for more details of the test.

Alert level B

```
PLAT971_ALERT_2_B Check Calcd Resid. Dens.  1.08Ang From C111          2.99 eA-3
```

Author Response: The residual density peak could not be modelled as any chemically sens.

Alert level C

```
PLAT221_ALERT_2_C Solv./Anion  Resd 2  F   Ueq(max)/Ueq(min) Range        4.2 Ratio
PLAT230_ALERT_2_C Hirshfeld Test Diff for    O02N     --C036     .         5.2 s.u.
PLAT234_ALERT_4_C Large Hirshfeld Difference F03I     --C04M     .        0.17 Ang.
PLAT234_ALERT_4_C Large Hirshfeld Difference C03V     --C04K     .        0.16 Ang.
PLAT234_ALERT_4_C Large Hirshfeld Difference C03V     --C040     .        0.18 Ang.
PLAT234_ALERT_4_C Large Hirshfeld Difference C04G     --C041     .        0.17 Ang.
PLAT234_ALERT_4_C Large Hirshfeld Difference C04T     --C040     .        0.16 Ang.
PLAT234_ALERT_4_C Large Hirshfeld Difference C04Q     --C042     .        0.16 Ang.
PLAT234_ALERT_4_C Large Hirshfeld Difference F04I     --C03A     .        0.20 Ang.
PLAT241_ALERT_2_C High   'MainMol' Ueq as Compared to Neighbors of       O02N Check
PLAT242_ALERT_2_C Low    'MainMol' Ueq as Compared to Neighbors of       C036 Check
PLAT342_ALERT_3_C Low Bond Precision on  C-C Bonds ...............    0.01313 Ang.
PLAT362_ALERT_2_C Short  C(sp3)-C(sp2) Bond  C03Z     - C04E     .        1.39 Ang.
PLAT362_ALERT_2_C Short  C(sp3)-C(sp2) Bond  C04J     - C049     .        1.40 Ang.
PLAT362_ALERT_2_C Short  C(sp3)-C(sp2) Bond  C04U     - C04D     .        1.40 Ang.
PLAT790_ALERT_4_C Centre of Gravity not Within Unit-Cell: Resd.  #           1 Note
              C35 H23 Ag3 F18 N6 O4
PLAT906_ALERT_3_C Large K Value in the Analysis of Variance ......      4.820 Check
PLAT906_ALERT_3_C Large K Value in the Analysis of Variance ......      2.554 Check
PLAT911_ALERT_3_C Missing FCF Refl Between Thmin & STh/L=    0.600        116 Report
              -15  1  0,   1  1  0, -15  2  0,   0  2  0, -11 21  0,  13-17  1,
               15 -4  1,  15 -3  1,  -1 -1  1,   1 -1  1,  -1  1  1, -14  5  1,
              -14 10  1,  -2 12  1, -13 18  1, -11 21  1,  14 -3  2,   0 -2  2,
               14 -2  2,  14 -1  2,   0  0  2,  14  0  2,  14  1  2,  14  2  2,
              -14  9  2,   9 14  2,   6 -9  3,   6 -8  3,  -6 -4  3,  14 -4  3,
                   (   86 More Missing: see the .ckf listing file)
PLAT971_ALERT_2_C Check Calcd Resid. Dens.  1.29Ang From C04K          2.46 eA-3
```

Author Response: The residual density peak could not be modelled as any chemically sens.

PLAT971_ALERT_2_C Check Calcd Resid. Dens. 1.07Ang From C041 2.30 eA-3

Author Response: The residual density peak could not be modelled as any chemically sens.

PLAT971_ALERT_2_C Check Calcd Resid. Dens. 1.03Ang From C04G 2.14 eA-3

Author Response: The residual density peak could not be modelled as any chemically sens.

PLAT971_ALERT_2_C Check Calcd Resid. Dens. 1.43Ang From O02N 2.04 eA-3

Author Response: The residual density peak could not be modelled as any chemically sens.

PLAT971_ALERT_2_C Check Calcd Resid. Dens. 1.12Ang From F031 1.97 eA-3

Author Response: The residual density peak could not be modelled as any chemically sens.

PLAT971_ALERT_2_C Check Calcd Resid. Dens. 1.06Ang From F04I 1.65 eA-3

Author Response: The residual density peak could not be modelled as any chemically sens.

PLAT971_ALERT_2_C Check Calcd Resid. Dens. 0.69Ang From C04W 1.59 eA-3

Author Response: The residual density peak could not be modelled as any chemically sens.

PLAT977_ALERT_2_C Check Negative Difference Density on H111 . -0.40 eA-3

Alert level G

```
CELLZ01_ALERT_1_G Difference between formula and atom_site contents detected.
CELLZ01_ALERT_1_G ALERT: check formula stoichiometry or atom site occupancies.
          From the CIF: _cell_formula_units_Z    6
          From the CIF: _chemical_formula_sum  C28.33 H16.33 Ag3 F18 N6 O2.67
          TEST: Compare cell contents of formula and atom_site data

          atom    Z*formula  cif sites diff
          C        169.98    170.00   -0.02
          H         97.98     98.00   -0.02
          Ag        18.00     18.00   -0.00
          F        108.00    108.00    0.00
          N         36.00     36.00    0.00
          O         16.02     16.00    0.02
PLAT002_ALERT_2_G Number of Distance or Angle Restraints on AtSite          3 Note
PLAT003_ALERT_2_G Number of Uiso or U(i,j)  Restrained non-H-Atoms        35 Report
PLAT041_ALERT_1_G Calc. and Reported SumFormula    Strings  Differ     Please Check
             Calc: C85 H49 Ag9 F54 N18 O8
             Rep.: C28.33 H16.33 Ag3 F18 N6 O2.67
PLAT042_ALERT_1_G Calc. and Reported MoietyFormula Strings  Differ     Please Check
             Calc: C35 H23 Ag3 F18 N6 O4, 2(C25 H13 Ag3 F18 N6 O2
             Rep.: 1.5(C10 H2 Ag2 F12 N4), 1.333(C10 H10 O2)
```

```
PLAT045_ALERT_1_G Calculated and Reported Z Differ by a Factor ...      0.333 Check
PLAT083_ALERT_2_G SHELXL Second Parameter in WGHT  Unusually Large      53.81 Why ?
PLAT154_ALERT_1_G The s.u.'s on the Cell Angles are Equal ..(Note)      0.001 Degree
PLAT171_ALERT_4_G The CIF-Embedded .res File Contains EADP Records          1 Report
PLAT172_ALERT_4_G The CIF-Embedded .res File Contains DFIX Records          9 Report
PLAT177_ALERT_4_G The CIF-Embedded .res File Contains DELU Records          5 Report
PLAT178_ALERT_4_G The CIF-Embedded .res File Contains SIMU Records          7 Report
PLAT186_ALERT_4_G The CIF-Embedded .res File Contains ISOR Records         13 Report
PLAT188_ALERT_3_G A Non-default SIMU Restraint Value has been used     0.0200 Report
PLAT188_ALERT_3_G A Non-default SIMU Restraint Value has been used     0.0200 Report
PLAT188_ALERT_3_G A Non-default SIMU Restraint Value has been used     0.0200 Report
PLAT188_ALERT_3_G A Non-default SIMU Restraint Value has been used     0.0200 Report
PLAT192_ALERT_3_G A Non-default DELU Restraint Value for SecondPar     0.0200 Report
PLAT192_ALERT_3_G A Non-default DELU Restraint Value for First Par     0.0010 Report
PLAT192_ALERT_3_G A Non-default DELU Restraint Value for SecondPar     0.0010 Report
PLAT242_ALERT_2_G Low    'MainMol' Ueq as Compared to Neighbors of       C04E Check
PLAT242_ALERT_2_G Low    'MainMol' Ueq as Compared to Neighbors of       C04J Check
PLAT242_ALERT_2_G Low    'MainMol' Ueq as Compared to Neighbors of       C04M Check
PLAT242_ALERT_2_G Low    'MainMol' Ueq as Compared to Neighbors of       C04O Check
PLAT242_ALERT_2_G Low    'MainMol' Ueq as Compared to Neighbors of       C04U Check
PLAT242_ALERT_2_G Low    'MainMol' Ueq as Compared to Neighbors of       C000 Check
PLAT242_ALERT_2_G Low    'MainMol' Ueq as Compared to Neighbors of       C02G Check
PLAT242_ALERT_2_G Low    'MainMol' Ueq as Compared to Neighbors of       C03A Check
PLAT242_ALERT_2_G Low    'MainMol' Ueq as Compared to Neighbors of       C03H Check
PLAT242_ALERT_2_G Low    'MainMol' Ueq as Compared to Neighbors of       C03J Check
PLAT242_ALERT_2_G Low    'MainMol' Ueq as Compared to Neighbors of       C03L Check
PLAT242_ALERT_2_G Low    'MainMol' Ueq as Compared to Neighbors of       C034 Check
PLAT242_ALERT_2_G Low    'MainMol' Ueq as Compared to Neighbors of       C01Y Check
PLAT242_ALERT_2_G Low    'MainMol' Ueq as Compared to Neighbors of       C02S Check
PLAT242_ALERT_2_G Low    'MainMol' Ueq as Compared to Neighbors of       C02W Check
PLAT242_ALERT_2_G Low    'MainMol' Ueq as Compared to Neighbors of       C03O Check
PLAT242_ALERT_2_G Low    'MainMol' Ueq as Compared to Neighbors of       C03P Check
PLAT242_ALERT_2_G Low    'MainMol' Ueq as Compared to Neighbors of       C039 Check
PLAT300_ALERT_4_G Atom Site Occupancy of Ag1        Constrained at        0.9 Check
PLAT300_ALERT_4_G Atom Site Occupancy of Ag2        Constrained at        0.9 Check
PLAT300_ALERT_4_G Atom Site Occupancy of Ag3        Constrained at        0.9 Check
PLAT300_ALERT_4_G Atom Site Occupancy of Ag4        Constrained at        0.1 Check
PLAT300_ALERT_4_G Atom Site Occupancy of Ag5        Constrained at        0.1 Check
PLAT300_ALERT_4_G Atom Site Occupancy of Ag6        Constrained at        0.1 Check
PLAT301_ALERT_3_G Main Residue  Disorder ..............(Resd    1)         5% Note
PLAT434_ALERT_2_G Short Inter HL..HL Contact F00I      ..F00R     .      2.84 Ang.
                                               1+x,y,1+z  =      1_656 Check
PLAT434_ALERT_2_G Short Inter HL..HL Contact F00Q      ..F012     .      2.71 Ang.
                                             -1+x,y,-1+z  =      1_454 Check
PLAT434_ALERT_2_G Short Inter HL..HL Contact F00R      ..F00X     .      2.84 Ang.
                                                 1+x,y,z  =      1_655 Check
PLAT434_ALERT_2_G Short Inter HL..HL Contact F00Y      ..F02L     .      2.76 Ang.
                                                   x,y,z  =      1_555 Check
PLAT434_ALERT_2_G Short Inter HL..HL Contact F02X      ..F02X     .      2.84 Ang.
                                             3-x,2-y,2-z  =      2_877 Check
PLAT606_ALERT_4_G Solvent Accessible VOID(S) in  Crystal Structure          ! Info
PLAT720_ALERT_4_G Number of Unusual/Non-Standard Labels ..........        215 Note
              Ag01    Ag02    Ag03    Ag04    Ag05    Ag06    F00A    F00B
              F00C    F00D    F00E    F00F    F00G    F00H    F00I    F00L
              F00P    F00Q    F00R    F00T    F00V    F00X    F00Y    F00Z
              F01B    F01C    F01H    F01R    F01T    F01W    F02E    F02F
              F02T    F02V    F04I    F010    F012    F013    F015    F019
```

```
              F026    F031    N00J    N00K    N00M    N00S    N00U    N00W
              N01A    N01E    N01F    N014    N016    N018    C01J    C01U
              C01V    C01Y    C01Z    H01Z    C02D    C02G    C02H    C02I
              H02I    C02Q    C02S    C02W    C02Z    C03A    C03C    H03C
              C03D    C03F    H03F    C03H    C03J    C03K    C03L    C03O
              C03P    C03Q    C022    C027    H027    C029    H029    C030
              C034    C039    F01O    F01P    F01S    F01X    F02J    F02L
              F02M    F02P    F02R    F02U    F02X    F03I    F03S    F03Y
              F020    F038    F043    F045    O02N    O017    N01K    N01N
              N02A    C03Z    C04R    H04R    C04F    C03V    H03A    H03B
              C04B    C04E    C04H    C04J    C04K    C04M    C04O    C04P
              H04P    C04U    C04W    H04W    C04G    H04G    C041    C04T
              C036    C000    C040    H04B    H04C    C047    H04D    H04E
              H04F    O00N    O01Q    C024    C03B    H03D    H03E    C03M
              H03M    C03N    H03N    C03T    C04L    H04H    H04I    H04J
              C028    C032    H03G    H03H    C033    C044    H044    O00O
              O021    C03E    C03U    C03X    H03I    H03J    C04C    C04N
              H04N    C04Q    H04Q    C035    C042    H042    C046    H04K
              H04L    H04M    C048    H04O    H04S    O01D    O011    C02B
              C02O    H02O    C02Y    H02A    H02B    C03G    H03K    C03R
              H03R    C03W    C04S    H04T    H04U    H04V    C023    C025
              C037    H03L    H03O    C04D    C04A    H04A    C049
PLAT779_ALERT_4_G Suspect or Irrelevant (Bond) Angle(s) in CIF ...      43.40 Deg.
              AG4  -N02A -N1      1_555   1_555   1_555 ......     #  195 Check
PLAT779_ALERT_4_G Suspect or Irrelevant (Bond) Angle(s) in CIF ...      13.00 Deg.
              N02A -C03Z -AG4     1_555   1_555   1_555 ......     #  197 Check
PLAT779_ALERT_4_G Suspect or Irrelevant (Bond) Angle(s) in CIF ...       6.80 Deg.
              N1   -C04F -AG4     1_555   1_555   1_555 ......     #  209 Check
PLAT779_ALERT_4_G Suspect or Irrelevant (Bond) Angle(s) in CIF ...       8.80 Deg.
              N01K -C04B -AG5     1_555   1_555   1_555 ......     #  224 Check
PLAT779_ALERT_4_G Suspect or Irrelevant (Bond) Angle(s) in CIF ...       7.80 Deg.
              N01N -C04H -AG5     1_555   1_555   1_555 ......     #  236 Check
PLAT779_ALERT_4_G Suspect or Irrelevant (Bond) Angle(s) in CIF ...      10.30 Deg.
              N9   -C04D -AG6     1_555   1_555   1_555 ......     #  434 Check
PLAT779_ALERT_4_G Suspect or Irrelevant (Bond) Angle(s) in CIF ...      11.20 Deg.
              N13  -C049 -AG6     1_555   1_555   1_555 ......     #  445 Check
PLAT790_ALERT_4_G Centre of Gravity not Within Unit-Cell: Resd.  #         3 Note
              C25 H13 Ag3 F18 N6 O2
PLAT860_ALERT_3_G Number of Least-Squares Restraints ............        369 Note
PLAT868_ALERT_4_G ALERTS Due to the Use of _smtbx_masks Suppressed         ! Info
PLAT883_ALERT_1_G Absent Datum for _atom_sites_solution_primary ..     Please Do !
PLAT910_ALERT_3_G Missing FCF Reflection(s) Below Theta(Min)[Deg]=       2.54 Note
                0  1  0,   0  0  1,
PLAT912_ALERT_4_G Missing # of FCF Reflections Above STh/L=  0.600       1003 Note
PLAT913_ALERT_3_G Missing # of Very Strong Reflections in FCF ....          3 Note
                2  0  3,   0 -4  4,   0 -4  5,
PLAT933_ALERT_2_G Number of HKL-OMIT Records in Embedded .res File         59 Note
              -16 -1 12, -16 -1 13, -16  0  9, -16  0 12, -16  0 13, -16  0 15,
              -16  1  7, -16  1  9, -16  1 10, -16  2  7, -16  2  9, -16  2 10,
              -16  2 15, -16  3  5, -16  3  6, -16  3  7, -16  3  9, -16  3 10,
              -16  4  9, -15 -2  7, -15  0  4, -15  2  7, -15 14  0, -14 -3  7,
              -14  5  1, -14  9  2, -14 10  1, -13-11  4, -13 -6 15, -13 -6 16,
              -13 -5 15, -13 -5 16, -13 -4 16, -13 -4 17, -12 -8 14, -12 -8 15,
              -12 -7 15, -12 -6 14, -12 -6 15, -11 -9 12, -11 -9 13, -11 -8 12,
              -11 -7 14, -10-10 10, -10-10 11, -10 -9 11,  -9-16  9,  -8-16  7,
                0  0  4,   0  2  3,
PLAT941_ALERT_3_G Average HKL Measurement Multiplicity ..........        2.6 Low
```

```
PLAT969_ALERT_5_G The 'Henn et al.' R-Factor-gap value ..........      3.655 Note
              Predicted wR2: Based on SigI**2  5.12 or SHELX Weight 17.09
PLAT978_ALERT_2_G Number C-C Bonds with Positive Residual Density.         1 Info
```

```
   0 ALERT level A = Most likely a serious problem - resolve or explain
   1 ALERT level B = A potentially serious problem, consider carefully
  27 ALERT level C = Check. Ensure it is not caused by an omission or oversight
  71 ALERT level G = General information/check it is not something unexpected

   7 ALERT type 1 CIF construction/syntax error, inconsistent or missing data
  44 ALERT type 2 Indicator that the structure model may be wrong or deficient
  16 ALERT type 3 Indicator that the structure quality may be low
  31 ALERT type 4 Improvement, methodology, query or suggestion
   1 ALERT type 5 Informative message, check
```

Validation response form

Please find below a validation response form (VRF) that can be filled in and pasted into your CIF.

```
# start Validation Reply Form
_vrf_PLAT221_lcz030
;
PROBLEM: Solv./Anion  Resd 2  F   Ueq(max)/Ueq(min) Range        4.2 Ratio
RESPONSE: ...
;
_vrf_PLAT230_lcz030
;
PROBLEM: Hirshfeld Test Diff for    O02N     --C036     .        5.2 s.u.
RESPONSE: ...
;
_vrf_PLAT234_lcz030
;
PROBLEM: Large Hirshfeld Difference F03I     --C04M     .       0.17 Ang.
RESPONSE: ...
;
_vrf_PLAT241_lcz030
;
PROBLEM: High   'MainMol' Ueq as Compared to Neighbors of       O02N Check
RESPONSE: ...
;
_vrf_PLAT242_lcz030
;
PROBLEM: Low    'MainMol' Ueq as Compared to Neighbors of       C036 Check
RESPONSE: ...
;
_vrf_PLAT342_lcz030
;
PROBLEM: Low Bond Precision on  C-C Bonds ..............    0.01313 Ang.
RESPONSE: ...
;
_vrf_PLAT362_lcz030
;
PROBLEM: Short  C(sp3)-C(sp2) Bond  C03Z     - C04E     .       1.39 Ang.
RESPONSE: ...
;
```

```
_vrf_PLAT790_lcz030
;
PROBLEM: Centre of Gravity not Within Unit-Cell: Resd.  #          1 Note
RESPONSE: ...
;
_vrf_PLAT906_lcz030
;
PROBLEM: Large K Value in the Analysis of Variance ......      4.820 Check
RESPONSE: ...
;
_vrf_PLAT911_lcz030
;
PROBLEM: Missing FCF Refl Between Thmin & STh/L=    0.600        116 Report
RESPONSE: ...
;
_vrf_PLAT977_lcz030
;
PROBLEM: Check Negative Difference Density on H111      .      -0.40 eA-3
RESPONSE: ...
;
# end Validation Reply Form
```

It is advisable to attempt to resolve as many as possible of the alerts in all categories. Often the minor alerts point to easily fixed oversights, errors and omissions in your CIF or refinement strategy, so attention to these fine details can be worthwhile. It is up to the individual to critically assess their own results and, if necessary, seek expert advice.

PLATON version of 17/09/2025; check.def file version of 17/09/2025

---

duplicate check

No duplication found

Datablock lcz030 - ellipsoid plot

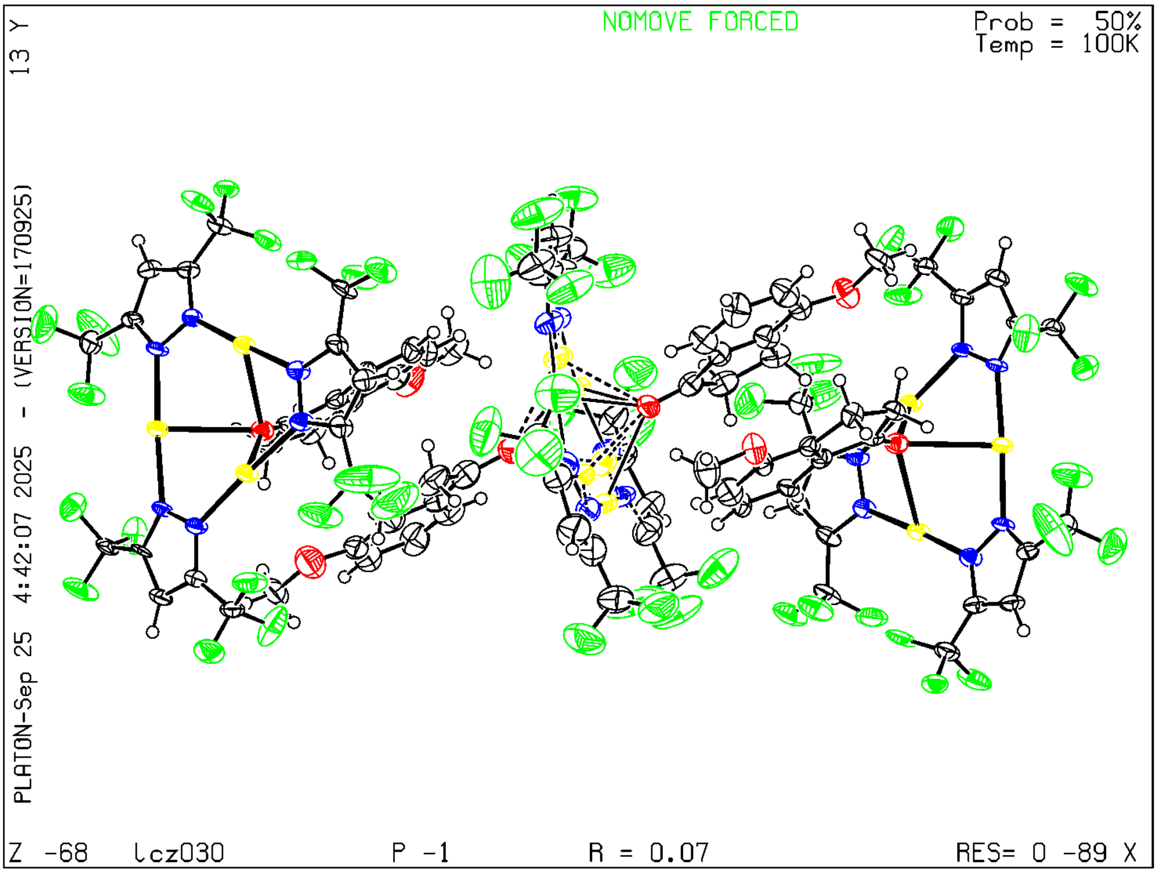

Supplement: Supplementary file 2 — Supporting File 2: anie72462‐sup‐0002‐DataFile.zip. [file ANIE-65-e1218503-s002.zip › supplementary_materials_crystallographic_data submit.zip!/cif_and_checkcif(1-114)/Ag3Pz3_30/Ag3Pz3_30_checkcif.pdf]
